# The interplay between bile acids and mucosal adaptive immunity

**DOI:** 10.1371/journal.ppat.1011356

**Published:** 2023-06-22

**Authors:** Ahmed Dawood Mohammed, Ryan A. W. Ball, Jason L. Kubinak

**Affiliations:** Department of Pathology, Microbiology, Immunology, University of South Carolina School of Medicine, Columbia, South Carolina, United States of America; Duke University School of Medicine, UNITED STATES

## Introduction

The fundamental role of the gut mucosal immune system is to maintain tolerance towards luminal antigens and this is achieved through a heavily coordinated and multilayered system of two-way interactions between gut-resident immune cells and molecular signals provided by the microbiome. Mucosal humoral immune responses (and primarily secretory IgA) are a major means by which the host regulates the taxonomic composition [1–7] spatial organization [8–10] and metabolic function of the microbiota [11–13]. One of the most important metabolic functions carried out by commensal microbes is biotransformation of host bile acids (BAs) [14].

BAs are host-derived amphipathic molecules that act as emulsifiers to promote the solubilization and absorption of dietary lipids and lipid-soluble vitamins [15]. BAs are mainly produced in the liver as primary BAs using cholesterol as a precursor and are then transported and stored in the gallbladder until being secreted into the duodenum postprandially. Approximately 95% of all BAs secreted into the gut will be reabsorbed in the distal ileum [16,17]. Under steady-state conditions, the 5% of BAs that escape this recycling process will reach the colon where they are modified by commensal gut bacteria to become secondary BAs. The gut microbiota modifies luminal BA biochemistry through different enzymatic reactions: deconjugation, dehydroxylation, dehydrogenation, epimerization, and oxidation-reduction. The first and rate-limiting step in bacterial BA biotransformation is the cleavage of glycine or taurine from BAs (deconjugation) and this is carried out by bacterial *bile salt hydroxylase* (*bsh*) enzymes. Bacterial deconjugation of BAs blocks the active transport of BAs through the apical sodium BA transporter (ASBT) [18]. Genetic studies of the human gut microbiota have revealed that members of all major bacterial phyla possess *bsh* genes and are capable of performing BA deconjugation [19,20]. In contrast to deconjugation, species within several families of the Firmicutes phylum (e.g., Lactobacillaceae, Clostridiaceae, Lachnospiraceae, Ruminococcaceae) appear to be primarily responsible for subsequent enzymatic reactions [21,22]. In addition, intestinal microbiota may regulate the synthesis of BAs in the liver through their ability to directly influence the balance of conjugated BAs in the lumen [23]. Micromolar shifts in the concentrations of hydrophobic BAs can stimulate enterocyte apoptosis [24,25], so enterohepatic circulation of BAs is a tightly regulated process operating through negative feedback mechanisms that maintain physiologically benign BA composition and concentrations. Recently, BAs have been described as signaling molecules that serve as ligands to the nuclear farnesoid X receptor (FXR) and the Takeda G protein-coupled receptor (TGR5) [26].

In addition to their contributions to host metabolism, BAs are also emerging as critical regulators of mucosal immune responses [27–31] with implications in various disease states [32–34]. How BAs promote immune tolerance in the gut, and whether/how the adaptive immune system operates to reinforce this, is an important emerging area of research in mucosal immunology. This review seeks to provide a brief overview of the current state of our knowledge regarding the two-way interaction between BAs and adaptive immunity, with an emphasis on recent work supporting that adaptive (humoral) immunity may serve to reinforce BA-induced immunological tolerance.

### Humoral immunity may regulate bacterial BA metabolism to reinforce immunological tolerance

Dysregulation of BA metabolism in the gut has been linked to numerous inflammatory and metabolic diseases in humans [35–38]. Studies by our group and others have shown that several different strains of antibody-deficient mice develop defects in lipid metabolism in the gut [3,39,40], which BAs profoundly influence. However, only recently has a direct link between B cells and the regulation of bacterial BA metabolism been shown. Using a CD19^−/−^ mouse model of spontaneous SI enteropathy that our group characterized [3], we were able to provide the first demonstration of this principle. In this study, we showed that the severity of SI enteropathy was sensitive to the bioavailability of BAs and that transfer of WT B cells was able to reduce bacterial overgrowth and alter BA biochemistry leading to a reduction in enteropathy. Additionally, altering BA bioavailability through supplementation with TUDCA, a highly absorbable BA, also reduced enteropathy.

**Fig 1:**
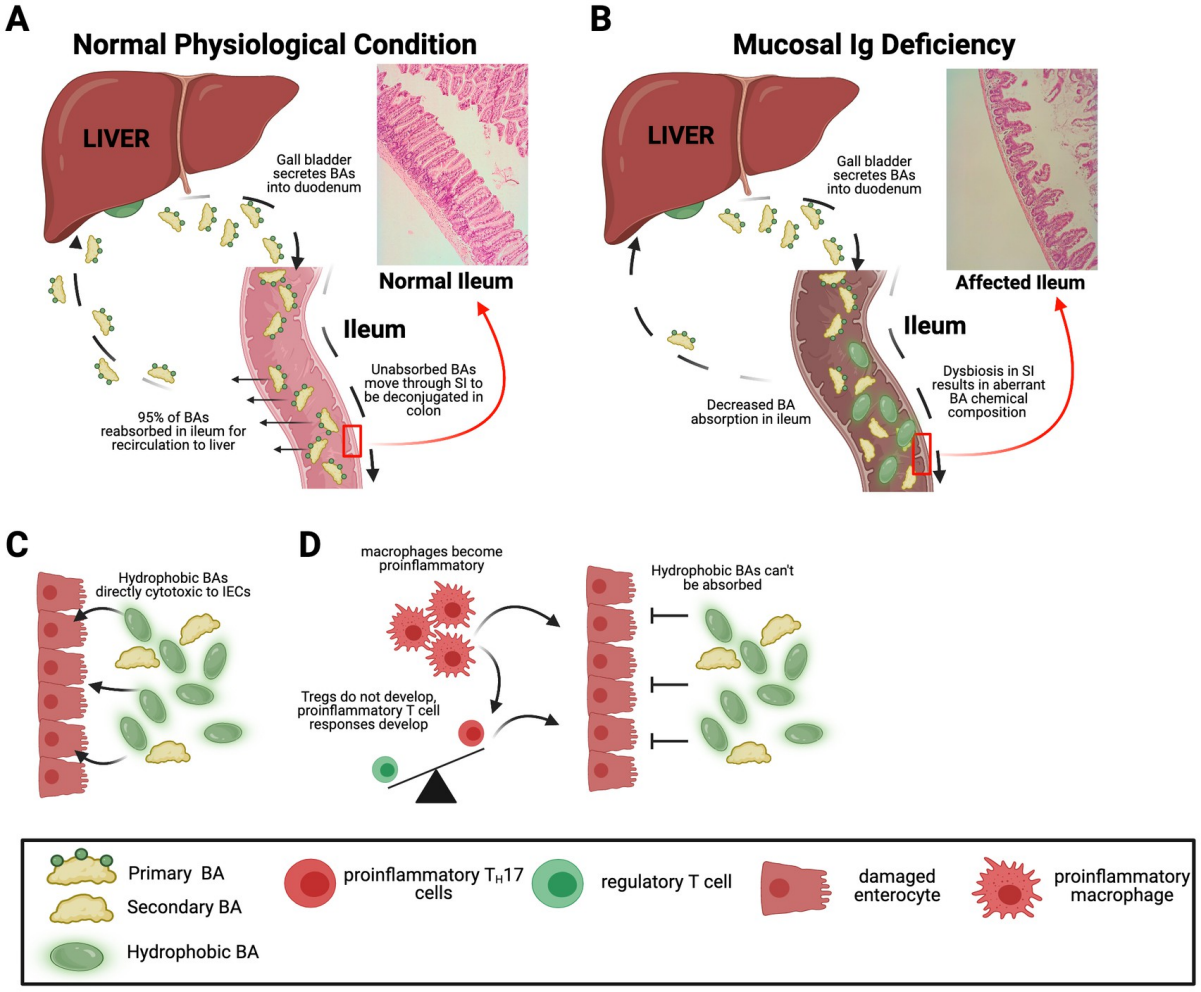
How humoral immunity may promote gut homeostasis through regulation of BA biochemistry in the gut. **(A)** Enterohepatic circulation of BAs under steady-state conditions. The vast majority of BAs are reabsorbed in the ileum for recirculation back to the liver. The remaining BAs (approximately 5% of the total pool) pass to the colon where bacterial metabolism leads to formation of secondary BAs. **(B)** In mucosal antibody deficiency, bacterial overgrowth in SI may result in aberrant bacterial BA metabolism that promotes SI enteropathy. Altered BA pools may be directly cytotoxic to ileal enterocytes **(C)**, and/or may be less absorbable leading to chronic inflammatory responses in the gut that promote degradation of the ileal lining **(D)**. Figure generated using Biorender.

Previously, we demonstrated that CD19^−/−^ and JH^−/−^ mice present with SIBO (which we defined as both total increases in bacterial biomass in the SI as well as altered taxonomic composition) [41,42]. We also demonstrated that this was associated with defects in bacterial BA metabolism [41]. To determine this, we performed 2 analyses. First, we demonstrated that transfer of WT B cells into CD19^−/−^ mice reduced the abundance of bacterial *bile salt hydrolase* (*bsh*) gene copies (specifically in the SI). *Bsh* catalyzes the first and rate-limiting step in bacterial BA metabolism and is widely conserved across bacterial clades [19,20]. Next, by depleting the microbiota of CD19^−/−^ mice with antibiotics and then colonizing cohorts of these mice with one of 2 isogenic strains of the model commensal bacterial species *Bacteroides thetaiotamicron* (*B*. *theta*) which either have intact BA-metabolizing capabilities (WT *B*. *theta*) or not (Δ*bsh B*. *theta*). We found that colonization of CD19^−/−^ mice with Δ*bsh B*. *theta* (but not WT *B*. *theta*) significantly altered SI BA pools as measured by UPLC-MS. As expected, ablation of the ability to metabolize BAs by *B*. *theta* resulted in a general shift in favor of conjugated BAs in Δ*bsh B*. *theta*-colonized mice. These changes to BA homeostasis were also associated with significant reductions in the severity of SI enteropathy. Collectively, these results indicate that enhanced bacterial BA metabolism in the SI may be the driver of disease in the context of B cell deficiency and that B cells directly impact BA homeostasis in the gut (Fig 1).

### BAs promote tolerogenic T cell responses in the gut

BAs serve as signaling molecules capable of inducing tolerogenic responses in innate immune cells. For example, signaling through the BA-sensing receptors FXR and TGR5 have been shown to tolerize gut macrophages to chronic LPS stimulation [27,43] and induce differentiation of IL-12 hypo-producing dendritic cells [44]. More recent work has also shown that BAs are capable of promoting tolerogenic adaptive immune responses. For example, lithocholic acid (a novel BA species generated by bacterial biotransformation of host-derived chenodeoxycholic acid) has been shown to limit TH1 and TH17 cell development and promote Treg development in the gut by regulating the transcription factors FoxP3 and Rorγt [30,31]. Recently, through the intracellular BA-sensing receptor CAR, BAs have also been shown to suppress TH17 cell development (in the SI specifically) through induction of T-cell-intrinsic IL10 production [29]. Most recently, Paik and colleagues demonstrated that bacterial modification of lithocholic acid (LCA), a human dominant secondary BA, inhibits T_H_17 cell differentiation in the small intestine [22]. Importantly, in the same study, multi-omics analysis of 2 groups of IBD patients also revealed that 3-oxolithocholic and isolithocholic acid, and the bacterial gene encoding the enzyme producing these 2 BAs, were negatively correlated with a T_H_17 transcriptional profile in these patients [22]. This was also demonstrated by Hang and colleagues where LCA metabolites 3-oxoLCA and isoalloLCA showed anti-inflammatory properties, suppressing T_H_17 differentiation and enhancing Treg development both in vivo and in vitro [30]. Finally, it has also recently been demonstrated that manipulation of bacterial BA metabolism influences adaptive immune tolerance induction in the gut. Specifically, the Kasper lab revealed that disrupting bacterial BA metabolism prohibited the development of an Rorγ+ subset of regulatory T cells (Rorγ+ Tregs) in the colon [28]. While anticipated, it is unknown whether mucosal antibodies influence bacterial BA metabolism in the gut lumen or how this influences mucosal immunological tolerance, but collectively these observations point to a role of humoral immunity to regulate bacterial BA metabolism which in turn influences tolerogenic T cell responses in the gut.

## Concluding remarks

“Small intestinal bacterial overgrowth” (SIBO) has been associated with dysregulated BA metabolism/circulation [45], and inflammatory diseases with SI-specific involvement, including Crohn’s disease [46] and celiac disease [47]. Notably, common variable immunodeficiency (CVID), the most commonly observed form of primary antibody deficiency, has also been associated with SI enteropathy [48], SIBO [49], and more recently, BA malabsorption [50]. Studies have also shown evidence of altered BA metabolites in the context of IBD. For example, in the Paik and colleagues study mentioned above, the authors also showed that 3-oxolithocholic acid and isolithocholic acid (the metabolites of the SBA lithocholic acid), as well as the gene encoding the rate-limiting enzyme of their production, were reduced in IBD patients [22]. In addition, another study by Duboc and colleagues showed that IBD patients with active disease had low secondary BA concentrations and an increase in conjugated BAs compared with healthy controls [51]. Furthermore, untargeted metabolomic and shotgun metagenomic analysis revealed that IBD patients had an increase in primary BAs and a reduction in secondary BAs compared to healthy controls [52]. Because most BAs are reabsorbed in the distal ileum, this tissue may be particularly sensitive to shifts in luminal BA biochemistry. Indeed, several lines of evidence support this. First, several different animal models (dogs, rabbits, rats, guinea pigs, and mice) have demonstrated that enhanced bacterial BA deconjugation is associated with reduced capacity to absorb BAs and the development of SI enteropathy, though mechanisms of pathogenesis remain undefined [53].

Within the last 2 decades, human and animal studies have clearly defined a role for mucosal humoral immunity in reenforcing benign physiological outcomes of host–microbiota interactions by managing the composition, spatial organization, and function of gut microbes. BAs are emerging as important tolerogenic signaling molecules with implications in various diseases ranging from enteropathy to metabolic and neurological disorders. The host factors regulating bacterial BA metabolism are poorly understood, and this represents a major gap in our understanding of how immunological tolerance develops and is reinforced in the gut. Recent evidence supports that BAs skew towards tolerogenic T cell responses in the gut and that humoral adaptive immunity may directly regulate bacterial BA metabolism to reinforce this phenotype.
